# Enhanced Fully Generalized Spatial Modulation for the Internet of Underwater Things

**DOI:** 10.3390/s19071519

**Published:** 2019-03-28

**Authors:** Zeyad A. H. Qasem, Hamada Esmaiel, Haixin Sun, Junfeng Wang, Yongchun Miao, Sheraz Anwar

**Affiliations:** 1 School of Information Science and Engineering, Xiamen University, Xiamen 316005, China; zeyadqasem@stu.xmu.edu.cn (Z.A.H.Q.); ycmiao@stu.xmu.edu.cn (Y.M.); sheraz@stu.xmu.edu.cn (S.A.); 2 Electrical Engineering Department, Faculty of Engineering, Aswan University, Aswan 81542, Egypt; h.esmaiel@aswu.edu.eg; 3 Department of Information and Communication Engineering, School of Electrical and Electronic Engineering, Tianjin University of Technology, Tianjin 300383, China; jfwang@tjut.edu.cn

**Keywords:** IoUT, spectral efficiency, energy efficiency, spatial modulation, FGSM

## Abstract

A full design of the Internet of Underwater Things (IoUT) with a high data rate is one of the greatest underwater communication difficulties due to the unavailability of a sustainable power source for the battery supplies of sensor nodes, electromagnetic spread weakness, and limited acoustic waves channel bandwidth. This paper presents a new energy-efficient communication scheme named Enhanced Fully Generalized Spatial Modulation (EFGSM) for the underwater acoustic channel, where the different number of active antennas used in Fully Generalized Spatial Modulation (FGSM) is combined with multiple signal constellations. The proposed EFGSM enhances energy efficiency over conventional schemes such as spatial modulation, generalized spatial modulation, and FGSM. In order to increase energy and spectral performance, the proposed technique conveys data bits not just by the number of active antenna’s index as in the existing traditional FGSM, but also using the type of signal constellation to increase the data bit rate and improve power saving without increasing the receiver’s complexity. The proposed EFGSM uses primary and secondary constellations as indexes to carry information, they are derived from others by geometric interpolation signal space. The performance of the suggested EFGSM is estimated and demonstrated through Monte Carlo simulation over an underwater acoustic channel. The simulation results confirm the advantage of the suggested EFGSM scheme not just regarding energy and spectral efficiency but also concerning the average bit error rate (ABER).

## 1. Introduction

Nearly 71% of the Earth’s surface is covered by the ocean, a connected body of water that is usually split into different principal oceans and miniature seas. Ocean warmth influences environments and weather patterns that change life on earth. Freshwater in lakes and rivers represents less than 1% of global surface area. The health of the ocean determines the health of the planet. The Internet of Underwater Things (IoUT) is described as a global network of intelligent, interconnected underwater things that allows controlling large unexplored water areas [1]. These things can be underwater sensors, autonomous surface vehicles (ASVs), ships, autonomous underwater vehicles (AUVs), etc. The IoUT can be very useful in many practical applications such as environmental monitoring, marine research and disaster prevention. Also, it could be a key technology in implementing a future smart underwater city. The IoUT was discussed in [2], and its importance was emphasized in previous work [2,3,4].

Mainly, four communication topologies can be used in IoUT [5,6]: optical, electromagnetic, magnetic induction and acoustic wave. Due to natural spreading problems such as scattering and line of sight (LOS), optical underwater communication its applications are restricted to be used only in clean water. Harsh underwater conductivity attenuates the electromagnetic and magnetic induction waves and restricts its operating frequency to be at a very low-frequency band. Acoustic is the widest topology used in underwater applications, where it achieves a wide coverage range which can be on the scale of kilometers, but unfortunately it is a high-power communication system with a limited channel bandwidth. The difference among these different physical underwater technologies in terms of features, pros and cons has been summarized in [6]. Power consumption represents the main IoUT challenge, due to the recharging capability problem of sensor nodes deployed in the underwater areas which suffer from the lack of a sustainable power source to feed their batteries. Many researchers have tried hard to increase energy and spectral efficiency in underwater/underground communication based on an effective magnetic induction physical layer technology [7,8,9,10,11]. They suggested magnetic induction communication as a physical layer for the internet of underwater/underground things [7,8,9,10,11] due to its shorter propagation delay and highly environment-independent channel behavior. However, unfortunately it still has many challenges to be applicable for the IoUT due to the short communication ranges of magnetic induction (10–100 m) compared to the km scale of acoustic communication. Also, its effective bandwidth and channel capacity are practically quite limited in most underwater transmission scenarios, within 100 KHz and 100 Kbps, respectively [7]. The reasons behind that are related to the sensitivity of a coil’s optimal operating frequency where small deviations can cause power reflections leading to a narrower effective bandwidth, and also to the operational limitation in low frequency in order to mitigate the high attenuation caused by eddy currents in the highly conductive water [11]. Hence, acoustic communication is still the most usable of the physical layer technologies which could be used in the IoUT. However, unfortunately according to the authors’ knowledge, there is no research which has addressed the IoUT energy saving problem in acoustic physical layer technologies regarding modulation techniques.

Multiple-input multiple-output (MIMO) technology represents the underwater communication key as it offers an increase in capacity and diversity gain for acoustic underwater wireless networks [12]. Unfortunately, MIMO has many limitations, such as implementation complexity with a high number of antennas, and its performance deterioration in multipath propagation environments such as the underwater channel. So, in many MIMO systems power loss problems lead to the implementation of a tiny number of transmitter antennas [13]. Hence, the spatial modulation (SM) scheme has been proposed where just a single transmitter antenna is activated in each communication, and a spatial domain is used to compensate the possible data rate [14].

SM is considered as one of the space modulation techniques (SMT), where one or more antennas can be selected for transmitting information. It provides a higher data rate than the traditional single-input-multiple-output (SIMO) systems, reduces its complexity without antenna synchronization, while avoiding inter-channel interference (ICI). Different SMTs have been introduced [13,15,16,17,18,19,20,21,22,23], extending the SMTs principles from enabling just one radio frequency (RF) chain at the transmitter side into enabling one or more RF chains with different techniques in order to achieve high throughput, enhanced signal-to-noise ratio (SNR), or both. One of these powerful techniques is generalized spatial modulation (GSM) [21,22]. In GSM, multiple antennas are active in each communication in order to improve the achievable data rate. Those activated antennas transmit the same symbols using the same data constellation for all symbols. Consequently, the incoming data is conveyed via the indices of the activated transmitter antennas and the modulated information symbol. Therefore, the available data rate of GSM is higher than that of the current SM because of the activation of a greater number of transmitter antennas. Based on the GSM mechanism, fully generalized spectral efficiency (FGSM) was proposed in [15,16], and the antenna subsets vary from only a single activated antenna, to multiple/all activated antennas. The variation of the number of transmitter antennas enhances the realization of the communication channel and therefore increases the available data rate.

As the data signal constellations can be used as an additional dimension to map the information bits [17], the transmitted data can be carried not just by the indices of the transmitter antennas as in SM, GSM, and FGSM but also by using the signal constellation type. The primary and secondary constellations are used to increase the number of combinations between modulations and transmitter antennas. A significant performance gain of spectral efficiency, receiver complexity reduction, and power saving are achieved in this way compared to conventional SMTs. Based on that, in this paper, a new scheme called enhanced fully generalized spatial modulation (EFGSM) is proposed. In this new scheme, the data signal constellation is used as an index, and information is conveyed through a varied number of transmitter antennas. At least two transmitter antennas are activated to transmit information. One or more are based on the primary signal constellation and other one or more are activated based on the secondary constellation. The numerical and simulation results show that a significant performance gain in terms of energy and spectral efficiency, receiver complexity and average bit error rate (ABER) is achieved using the proposed scheme compared to conventional FGSM, GSM, and SM. Our main contributions can be summarized as follows:

(1). We investigate for the first time the power consumption of the IoUT, and a new energy efficient communication system for the IoUT is proposed based on a modification of the fully generalized spatial modulation. The key idea of the proposed scheme uses the data signal constellations as an additional dimension to map the information bits by combining the enhanced SM and FGSM.
(2). Using the well-known union boundary technique, we determine closed-form ABER approximations for the proposed EFGSM in the acoustic IoUT. Furthermore, inclusive Monte Carlo simulations are applied to validate the derived formula.
(3). We calculate the power saved using the proposed EFGSM scheme in reference to the M quadrature amplitude modulation (M-QAM) and other SMTs which can be used as a modulation scheme in the acoustic IoUT.
(4). We analyze and evaluate the receiver’s computational complexity of the suggested EFGSM by defining the total number of real operations (TNRO) needed at their maximum likelihood (ML) decoders. Furthermore, it is compared with the computational complexity of the other current SMTs.

The rest of the paper is structured as follows: Section 2 represents the relevant literature. In Section 3, we explain the proposed EFGSM system model. The performance analysis of the EFGSM is introduced in Section 4. Section 5 presents the simulation results, and finally the paper is concluded in Section 6.

## 2. Related Work

In recent years, spatial modulation techniques (SMTs) such as SM [18,19], GSM [21,22], quadrature spatial modulation (QSM) [23], FGSM [15,16], and fully quadrature spatial modulation (FQSM) [16] have been considered as promising MIMO techniques that achieve better performances in terms of spectral and energy efficiency as well as in reducing system complexity in comparison with the conventional MIMO counterparts.

In SM [18,19] one single RF chain, out of the $N_{T}$ antennas, is activated to convey the data constellation symbol, and the index of this active RF chain carries additional data bits. The available data rate on the traditional SM technique $R_{SM}$ is relative to signal constellation M-ary and the number of transmitted antennas $N_{T}$. It allows poor communication since just one transmitter antenna is working at any given time. Consequently the SM achievable data rate, $R_{SM}$, can be expressed as follows [18,19]:

(1) $$R_{SM}=\;log_{2}\left(M\right)+\;log_{2}\left(N_{T}\right),$$

where *M* represents the modulation order of the conveyed symbol.

The GSM [21,22] was proposed to improve the SM achievable data rate by activating multiple antennas. In GSM, the same data is transmitted through two or more antennas using the same data signal constellation. However, the data rate improvement is marginal, moreover, activating more than one antenna without efficient use will rapidly consume the energy source. The GSM achievable data rate, $R_{GSM}$, is considered as proportional to the logarithm of the binomial coefficients, and can be written as follows [21,22]:

(2) $$R_{GSM}=\;log_{2}\left(M\right)+\;⎣log_{2}\left(\begin{array}{c} N_{T}\\ N_{u} \end{array}\right)⎦,$$

where $⎣\cdot ⎦,\;\left(\begin{array}{c} \cdot \\ \cdot \end{array}\right)\mathrm{and}\;N_{u}$ indicate the floor operator, the binomial coefficient, and the number of transmitting active antennas, respectively.

The spatial constellation diagram was extended into two orthogonal dimensions in QSM where real and imaginary parts are transmitted through one or more transmitter antenna [23]. The real part of the data constellation symbol is transmitted via one dimension, and the imaginary part is transmitted via another dimension. By this data bit transmission method, the achievable data rate is enhanced compared to SM and GSM, but regrettably, the enhanced data rate is logarithmic with the square of the number of $N_{T}$. Accordingly, the QSM achievable data rate, $R_{QSM}$ can be expressed as follows [23]:

(3) $$R_{QSM}=\;log_{2}\left(M\right)+\left(log_{2}N_{T}^{2}\right),$$

In enhanced SM (ESM) [13,17], the information bits are not only transmitted by the active transmitter antennas index(es) but also by using the signal constellation type. Hence, an integer of the power of two transmitter antenna $N_{T}$ out of the transmitting antennas can be selected to be used in information transmission. Half of the activated transmitter antennas are forced to transmit information based on the primary signal constellation, and the others transmit information using the secondary signal constellation which is obtained by a single-step geometric insertion among the points of the primary signal constellation. The points of the secondary constellation are located at the midpoints of the intersections created by neighbor points of the M-QAM which are used as a primary constellation [17]. For example, if there are four transmitter antennas used, the maximum number of potential working antennas is two to transmit antenna conveyed information, one activated by the primary signal constellation, and the other is activated by secondary signal constellation. The ESM marginally increases the achievable data rate with significant improvement in the average energy per transmitted symbol $\left(E_{s}\right)$.

In FGSM, the number of active transmitter antennas is used as an index to transmit data by the same data signal constellation [15,16] where the active antennas indexes are used to send information bits, and also their varying quantity r is used as an index to carry additional information bits. For example, one or more antennas can be activated to send data using the same data signal constellation, hence the number of activated antennas carries information as well. In other words, it is a combination of SM and GSM where if one antenna is activated, FGSM is similar to SM, while if multiple/all transmit antennas are activated, the FGSM is equivalent to GSM. In this way, FGSM achieves greater data bit transmission, and its achievable data rate $R_{FGSM}$ is given by:

(4) $$R_{FGSM}=\;log_{2}\left(M\right)+\;⎣log_{2}\overset{N_{t}}{\underset{k=1}{\sum }}\left(\begin{array}{c} N_{T}\\ k \end{array}\right)⎦=\;log_{2}\left(M\right)+\;N_{T}-1,$$

As in the FGSM combination technique, the varied number of active antennas has been applied to QSM in [16] resulting in FQSM. FQSM transmits the real and the imaginary components of the signal constellation in one or more transmitter antennas in orthogonal dimensions, and its achievable data rate $R_{FQSM}$ is expressed as follows:

(5) $$R_{FQSM}=\;log_{2}\left(M\right)+\;2⎣log_{2}\overset{N_{T}}{\underset{k=1}{\sum }}\left(\begin{array}{c} N_{T}\\ k \end{array}\right)⎦=\;log_{2}\left(M\right)+\;2(N_{T}-1).$$

Despite the achievable data rate enhancement in FGSM and FQSM compared with conventional schemes, transmitting the same data by multiple transmitter antennas increases the average energy per transmitted symbol which effects power saving dramatically.

## 3. Proposed EFGSM

**Figure 1:**
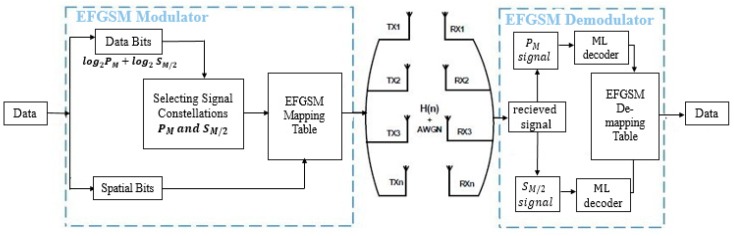
Proposed enhanced fully generalized spatial modulation (EFGSM).

The block diagram of the proposed EFGSM including the modulator and demodulator is shown in Figure 1. The incoming data bits are partitioned into two groups. The first group contains $log_{2}\left(P_{M}\right)+\;log_{2}\left(S_{M/2}\right)$ bits that are referred to as data bits, where $P_{M}$ and $S_{M/2}$ stand for primary and secondary signal constellations, respectively. This group is used for modulating the signal constellation symbol from two signal constellation diagrams as designed in ESM-Type1 in [17]. The second combination of bits represents the spatial bits; it is applied to choose the antenna subset utilized in transmitting information of the constellation symbol. The antenna subset of EFGSM differs from the case of activating just one transmitter antenna to the case of activating $N_{T}/2$ transmitter antennas for each the primary and secondary signal constellations. This contradicts the approaches of the current FGSM introduced in [15,16] and ESM-Type1 in [17], in which a varied transmitter antenna number is activated in the case of FGSM, and different signal constellations are used in case of ESM-Type1. The variation of signal constellations and transmitter antennas enhance the underwater acoustic channel utilization, reduces the average energy per transmitted symbol, and obviates the BER deterioration. The proposed EFGSM can be considered as a combination of ESM and FGSM. Therefore, the achievable data rate of the proposed EFGSM can be expressed as follows:

(6) $$R_{EFGSM}=\;log_{2}\left(P_{M}\right)+\;⎣log_{2}\sum_{k=2}^{N_{T}}\left(\begin{array}{c} N_{T}\\ k \end{array}\right)⎦+\;log_{2}\left(S_{M/2}\right),$$

where k represents the number of active antennas for both primary and secondary constellations.

Different subspaces based on different number of antennas utilize different combinations of active antennas. In all of these subspaces one/multiple active antennas transmit symbols based on the primary signal constellation, $P_{M}$, while the other active/multiple antennas transmit symbols based on the secondary signal constellation, $S_{M/2}$. The group of the spatial bits $⎣log_{2}\sum_{k=2}^{N_{T}}\left(\begin{array}{c} N_{T}\\ k \end{array}\right)⎦$, is used to select the combination of active antennas. Generally, we can say that the transmitted information bits are based on three groups: the primary signal constellation, the secondary signal constellation, and the active antenna indexes. First, the information bits are carried via the primary constellation with an achievable bit rate of $log_{2}\left(P_{M}\right)$. Secondly, the information bits are conveyed using the secondary signal constellation with an achievable bit rate of $⎣log_{2}\left(S_{M/2}\right)⎦$. Finally, the utilization of the spatial combination of antennas and modulation results in an achievable bit rate of $⎣log_{2}\sum_{k=2}^{N_{T}}\left(\begin{array}{c} N_{T}\\ k \end{array}\right)⎦$. Unlike the conventional FGSM, using primary and secondary signal constellations increases the number of antenna/constellation combinations. Therefore, EFGSM increases the achievable data rate and decreases energy consumption without altering system complexity. For example, in case of using 4-QAM as a primary signal constellation and binary phase shift keying (BPSK) as a secondary signal constellation, the gutted achievable data rate is 7 bit per channel used (bpcu), on the other hand, the achievable data rate of the conventional FGSM using the same MIMO configuration is only 5 bpcu. However, when using 8-QAM as a primary signal constellation and QPSK as a secondary signal constellation, with the same number of transmitter antennas, the achievable data rate will increase to 9 bpcu in the proposed EFGSM and only 6 bpcu in case of the conventional FGSM.

For a better explanation of the proposed EFGSM transmission procedure, assume, without loss of generality, $N_{T}$ = $N_{R}$ = 4 as shown in Figure 1, where $N_{R}$ represents the receiver antennas. Using 4-QAM modulator as a primary signal constellation type, the secondary signal constellation is equivalent to the throughput of half of the primary signal constellation, such as BPSK. In this case, the antenna indexing of the proposed EFGSM can be represented as shown in Table 1, for 7 bpcu. For illustration, in the example mentioned, consider the following generated bits, $\left[\begin{array}{cc} 010 & 1010 \end{array}\right]$, are needed to be transmitted. The first three bits [010] represents the bits carried via the primary and secondary signal constellations. [01] is equivalent to $log_{2}\left(P_{M}\right),$ and conveyed using the primary signal constellation, and the other part [0] (equal to the half of primary signal constellation bits), equivalent to $log_{2}\left(S_{M/2}\right)$, transmitted via the secondary signal constellation. The remaining four bits, [1010], are transmitted via the antenna index $T_{x1}T_{x4}$. The secondary signal constellation bits will be transmitted over the active antenna, $T_{x1}$, and primary signal constellation will be transmitted over the active transmitting antenna $T_{x4}$. Therefore, the $N_{T}\times 1$ transmitted vector of the EFGSM is $x=\left[\begin{array}{cc} S_{M/2} & \begin{array}{ccc} 0 & 0 & P_{M} \end{array} \end{array}\right]$. That vector of the EFGSM is sent via $N_{T}$x $N_{R}$ over an uncorrelated underwater acoustic channel, H contaminated by an additive white Gaussian noise with $n\sim CN\left(0,N_{o}\right)$.

The received signal $y\;\in \;{∁}^{N_{R}x1}$ at receiver side is given by:

(7) $$y=\;h_{l_{P}}S_{P_{M}}+\;\;h_{l_{S}}S_{S_{M/2}}+n,$$

and,

(8) $$h_{l_{P}}=\;\sum_{i=1}^{N_{l_{P}}}h_{i}\;\;,h_{l_{S}}=\;\sum_{k=1}^{N_{l_{S}}}h_{k},$$

where, $h_{l_{P}}$ and $h_{l_{S}}$ indicate the summation of columns of activated antennas channel required to transmit the primary signal constellation and secondary constellation, respectively. $N_{l_{P}}$, $N_{l_{S}}$ = 1, 2 … $⎣\frac{N_{T}}{2}⎦$ with ⎡.⎤ denotes the ceiling operator and $h_{i}$ denotes the $i^{th}$ columns of channel H.

The knowledge of the channel at the receiver side is assumed to be perfect. Therefore, the maximum-likelihood (ML) decoder can be used in the EFGSM system and is expressed as:

(9) $$\left[\begin{array}{cc} {\tilde{h}}_{\tilde{l_{P}}} & {\tilde{h}}_{\tilde{l_{S}}} \end{array}\;\;\;\begin{array}{cc} {\tilde{S}}_{P_{M}} & {\tilde{S}}_{S_{M/2}} \end{array}\right]=\arg\underset{h_{l_{P}},h_{l_{S}},\;\;S_{P_{M}},\;S_{S_{M/2}}}{\underbrace{min}}||y-\;h_{l_{P}}S_{P_{M}}-\;h_{l_{S}}S_{S_{M/2}}||{}^{2}.$$

In the ML decoder, the indexed information bits are recovered using the estimated antenna indices combination and the primary and secondary estimated data symbol constellations.

## 4. Performance Analysis of EFGSM for the Underwater Acoustic Channel

While the next generation of underwater technologies requires the support of the IoUT, due to the characteristics of underwater communication tools the idea of practical and robust transmission techniques for IoUT still faces significant difficulties. One of the challenges facing the IoUT is the difficulty of recharging the underwater nodes [15]. In order to ensure and facilitate better and continuous operation of IoUT nodes, the proposed EFGSM has been evaluated over the underwater acoustic channel as a low-energy MIMO modulation scheme. The primary goal of the proposed EFGSM is to minimize the power loss in underwater communication and increase the underwater communication spectral efficiency for IoUT applications. This section provides a mathematical framework for performance evaluation of the pairwise error probability (PEP), energy efficiency improvement and system complexity.

### 4.1. ABER Performance Analysis

As in the conventional SMTs, the ABER of the proposed EFGSM can be evaluated by using a well-known union boundary technique as in [15,16,24]:

(10) $$ABER\leq \;\frac{1}{2^{R}}\;\overset{2^{R}}{\underset{v=1}{\sum }}\overset{2^{R}}{\underset{k=1}{\sum }}\frac{N\left(x_{v}\to {\tilde{x}}_{k}\right)p_{r}\left(x_{v}\to {\tilde{x}}_{k}\right)}{R},$$

where $p_{r}\left(x_{v}\to {\tilde{x}}_{k}\right)$ represents the pairwise error probability (PEP) which defines the detection of erroneous symbol vector ${\tilde{x}}_{k}$ rather than the transferred symbol vector $x_{v}$. $N\left(x_{v}\to {\tilde{x}}_{k}\right)$ indicates how many bits in failure are within $x_{v}$ and ${\tilde{x}}_{k}$, while R is the mean of the achievable bit rate. Accordingly, the conditional pairwise error probability (C-PEP) can be given as follows:

(11) $$p_{r}\left(x_{v}\to {\tilde{x}}_{k}|\;H\right)=p_{r}\left(||y-\;h_{l}S_{j}||{}^{2}>||y-\;{\tilde{h}}_{\tilde{l}}{\tilde{S}}_{j}||{}^{2}\right),$$

where $h_{l}S_{j}=(h_{l_{P}}S_{P_{M}}+\;h_{l_{S}}S_{S_{M/2}}),$ is the received signal and includes both the primary and secondary constellations. Hence, using y as in (7), the reduced C-PEP can be written as:

(12) $$p_{r}\left(x_{v}\to {\tilde{x}}_{k}|H\right)=\;p_{r}\left({\left||n|\right|}^{2}>||n+\;h_{l}S_{j}-\;{\tilde{h}}_{\tilde{l}}{\tilde{S}}_{j}||{}^{2}\right).$$

Letting $z=\;e_{l}S_{j}$ and $\tilde{z}=\;e_{\tilde{l}}\tilde{S_{j}}$, the right-hand side of (12) can be rewritten after expanding the norm operator as follows:

(13) $$p_{r}\left(x_{v}\to {\tilde{x}}_{k}|\;H\right)=\;p_{r}\left({\left||n|\right|}^{2}>||n+\;H{\left(z\;-\;\tilde{z})|\right|}^{2}\right),$$

where z and $\tilde{z}$ $\in \;{\mathbb{R}}^{N_{T}\times 1}$ defining the columns of identity matrix $I_{N_{T}\times N_{R}}$. If we let $f=\left(z\;-\;\tilde{z}\right)$, (13) can be rewritten as follows:

(14) $$p_{r}\left(x_{v}\to {\tilde{x}}_{k}|\;H\right)=\;p_{r}\left(-2ℛ\left(n^{H}H\;f\right)>f^{H}H^{H}Hf\right),$$

where $ℛ\left(n^{H}Hf\right)$ defines a Gaussian random variable (RV) distributed as $ℛ\left(n^{H}Hf\right)\sim \left(0,\;\frac{\sigma_{v}{}^{2}\;f^{H}H^{H}Hf}{2}\right)$. Hence the expression of C-PEP in (14) can be rewritten as follows:

(15) $$p_{r}\left(x_{v}\to {\tilde{x}}_{k}/H\right)=\;p_{r}\left(-2\sqrt{\frac{\sigma_{v}{}^{2}\;f^{H}H^{H}Hf}{2\;}}u>\;f^{H}H^{H}Hf\right),$$

where *u* represents a regular Gaussian RV. The C-PEP in (15) can be rewritten after reducing it as:

(16) $$p_{r}\left(x_{v}\to {\tilde{x}}_{k}|H\right)=\;p_{r}\left(u>\;\;\sqrt{\frac{\sigma_{v}{}^{2}\;f^{H}H^{H}Hf}{2\;\sigma_{v}{}^{2}}}\right).$$

So,

(17) $$p_{r}\left(x_{v}\to {\tilde{x}}_{k}|H\right)=\;Q\left(\;\sqrt{\frac{\sigma_{v}{}^{2}\;f^{H}H^{H}Hf}{2\;\sigma_{v}{}^{2}}}\right).$$

Therefore, (17) can be represented as follows:

(18) $$p_{r}\left(x_{v}\to {\tilde{x}}_{k}|H\right)=\;E_{H}\left\{Q\left(\;\frac{d^{H}d}{2\;\sigma_{v}{}^{2}\;}\right)\right\},$$

where $E_{H}\left\{.\right\}$ indicates the expectation across the fading channel ***H***, we can express d as follows:

(19) $$d=Hf=\overset{N_{R}}{\underset{j=1}{\sum }}r_{j}f,$$

where $r_{j}$ is the $j^{th}$ row of the channel matrix ***H***. Moreover, the term $d^{H}d$ is chi-squared RV with a $2N_{R}$ degree of freedom (DOF). Hence, it can be written as a function of ψ as follows:

(20) $$d^{H}d=\;\frac{\sigma_{h}{}^{2}\;f^{H}f}{2}\;\psi ,$$

where ψ represents a standard chi-squared RV. Furthermore, the probability density function (PDF) of ψ can be calculated as:

(21) $$F\left(\psi \right)=\;\{\begin{array}{c} \frac{\psi^{N_{R}-1}\;e^{\frac{-\psi }{2}}}{2^{N_{R}}\;\Gamma \left(N_{R}\right)}\;\;\;\;\;\;\psi >0\;\\ 0\;\;\;\;\;\;\;\;\;\;\;\;\;otherwise \end{array},$$

where Γ(.) represents the public gamma function. From (18), (20) and (21), the PEP can be expressed as follows:

(22) $$p_{r}\left(x_{v}\to {\tilde{x}}_{k}\right)=\;\overset{\infty }{\underset{0}{\int }}Q\left(\sqrt{\frac{\sigma_{h}{}^{2}\;f^{H}f}{4\;\sigma_{v}{}^{2}}}\right)\frac{\psi^{N_{R}-1}\;e^{\frac{-\psi }{2}}}{2^{N_{R}}\;\Gamma \left(N_{R}\right)}\;d\psi .$$

The Q function can be alternatively expressed:

(23) $$Q\left(x\right)=\;\frac{1}{\pi }\overset{\frac{\pi }{2}}{\underset{0}{\int }}e^{\frac{x^{2}}{sin^{2}\theta \;}}d\theta .$$

As such, the PEP can be rewritten as follows:

(24) $$p_{r}\left(x_{v}\to {\tilde{x}}_{k}\right)=\;\frac{1}{2^{N_{R\;}}\pi \;\Gamma \left(N_{R}\right)}\;\overset{\frac{\pi }{2}}{\underset{0}{\int }}\overset{\infty }{\underset{0}{\int }}e^{-\left(\frac{1}{2}+\;\frac{B}{sin^{2}\theta }\right)\psi }\;\psi^{N_{R}-1}d\theta d\psi ,$$

where $B=\;\frac{\sigma_{h}{}^{2}\;f^{H}f}{4\;\sigma_{v}{}^{2}}.$ Define $I_{1}$ as:

(25) $$I_{1}=\;\overset{\infty }{\underset{0}{\int }}e^{-\left(\frac{1}{2}+\;\frac{B}{sin^{2}\theta }\right)\psi }\;\psi^{N_{R}-1}d\psi .$$

Then $I_{1}$ in (25) can be computed according to [16,24]:

(26) $$I_{1}=\;\frac{2^{N_{R}}\;\Gamma \left(N_{R}\right)}{{\left(\frac{sin^{2}\theta +B}{sin^{2}\theta }\right)}^{N_{R}}}.$$

Substituting (26) in (24), the PEP can be given as follows:

(27) $$p_{r}\left(x_{v}\to {\tilde{x}}_{k}\right)=\;\frac{1}{\pi }\overset{\frac{\pi }{2}}{\underset{0}{\int }}{\left(\frac{sin^{2}\theta }{sin^{2}\theta \;+B}\right)}^{N_{R}}d\theta .$$

The PEP in (28) can be expressed as follows:

(28) $$p_{r}\left(x_{v}\to {\tilde{x}}_{k}\right)=\;\frac{1}{2}\left[1-U\left(B\right)\overset{N_{R}-1}{\underset{k=0}{\sum }}\left(\begin{array}{c} 2k\\ k \end{array}\right){\left(\frac{1-\;U^{2}\left(B\right)}{4}\right)}^{k}\right],$$

where,

(29) $$U\left(B\right)=\;\sqrt{\frac{B}{1+B\;}.}$$

Furthermore, (29) can be rewritten as [24] as follows:

(30) $$p_{r}\left(x_{v}\to {\tilde{x}}_{k}\right)={\left[\frac{1-\;U\left(B\right)}{2}\right]}^{N_{R}}\overset{N_{R}-1}{\underset{k=0}{\sum }}\left(\begin{array}{c} N_{R}-1+k\\ k \end{array}\right){\left(\frac{1-\;U\left(B\right)}{2}\right)}^{k}.$$

Pair-wise error probability at high SNR is provided in (28) and (30), it can be expressed in an asymptotic form using a Taylor series (TS) as follows:

(31) $$p_{r}\left(x_{v}\to {\tilde{x}}_{k}\right)\;≅\;\left(\frac{1}{2}\right)\left(\frac{\Gamma \left(N_{R}+0.5\right)}{\sqrt{\pi }\;N_{R}!}\right){\left(\frac{1}{B}\right)}^{N_{R}}$$

Each type of SMT has a different PEP which essentially depend on B, where B can be expressed as follows:

(32) $$B=\;\frac{\sigma_{h}{}^{2}}{4\;\sigma_{v}{}^{2}}\varepsilon ,$$

where $\varepsilon =\;d^{H}d$ which can be specified based on the constellation symbols information utilized in the communication process of SMTs. For the proposed EFGSM, it is as defined in (33). In that expression $N_{P_{M}}$ and $N_{S_{M/2}}$ indicate the number of active antennas that are assigned to transmit the primary and secondary constellations of the data symbols, respectively. Moreover, ${\tilde{N}}_{{\tilde{P}}_{M}}$ and ${\tilde{N}}_{{\tilde{S}}_{M/2}}$ indicate the estimated number of antennas. $\Upsilon \left(P_{M},\;{\tilde{P}}_{\tilde{M}}\right)$ and $\Upsilon \left(S_{M/2},\;{\tilde{S}}_{\tilde{M}/2}\right)$ indicate the number of indices which are different between $P_{M},{\tilde{\;P}}_{\tilde{M}}$ and $S_{M/2},\;{\tilde{S}}_{\tilde{M}/2}$, respectively.

Proof, in the proposed EFGSM, any random value can represent a different number between $P_{M},\;{\tilde{P}}_{\tilde{M}}$ or between $S_{M/2},\;{\tilde{S}}_{\tilde{M}/2}$. The chosen number of active antennas convey the data symbol of the primary constellation or the secondary constellation, while the equivalent of the ML decoder estimated antennas number of the EFGSM receiver side is uncertain.

(33) $$\{\begin{array}{l} N_{P_{M}}{\left|S_{P_{M}}-\;{\tilde{S}}_{P_{M}}\right|}^{2}+\;N_{S_{\frac{M}{2}}}{\left|S_{S_{\frac{M}{2}}}-\;{\tilde{S}}_{S_{\frac{M}{2}}}\right|}^{2\;}\\ \;\;\;\;\;\;\;if\;h_{l_{P}}=\;{\tilde{h}}_{\tilde{l_{P}}},\;h_{l_{S}}=\;{\tilde{h}}_{\tilde{l_{S}}}\;\\ N_{P_{M}}{\left|S_{P_{M}}-\;{\tilde{S}}_{P_{M}}\right|}^{2}+\;\left[\frac{N_{S_{\frac{M}{2}}}+\;{\tilde{N}}_{{\tilde{S}}_{\frac{M}{2}}\;}-\;\Upsilon \left(S_{\frac{M}{2}},\;{\tilde{S}}_{\frac{\tilde{M}}{2}}\;\right)\;}{2}\right]{\left|S_{S_{\frac{M}{2}}}-\;{\tilde{S}}_{S_{\frac{M}{2}}}\right|}^{2\;}+\;\left[\frac{\Upsilon \left(S_{\frac{M}{2}},\;{\tilde{S}}_{\frac{\tilde{M}}{2}}\;\right)+N_{S_{\frac{M}{2}}}-\;{\tilde{N}}_{{\tilde{S}}_{\frac{M}{2}}\;}\;}{2}\right]{\left|S_{S_{\frac{M}{2}}}\right|}^{2\;}+\left[\frac{\;\Upsilon \left(S_{\frac{M}{2}},\;{\tilde{S}}_{\frac{\tilde{M}}{2}}\;\right)-N_{S_{\frac{M}{2}}}+\;{\tilde{N}}_{{\tilde{S}}_{\frac{M}{2}}\;}\;}{2}\right]{\left|{\tilde{S}}_{S_{\frac{M}{2}}}\right|}^{2\;}\\ \;\;\;\;\;\;\;if\;h_{l_{P}}=\;{\tilde{h}}_{\tilde{l_{P}}},\;h_{l_{S}}\neq \;{\tilde{h}}_{\tilde{l_{S}}}\\ N_{S_{\frac{M}{2}}}\left|S_{S_{\frac{M}{2}}}-\;{\tilde{S}}_{S_{\frac{M}{2}}}\right|+\;\left[\frac{N_{P_{M}}+\;{\tilde{N}}_{{\tilde{P}}_{M}}-\;\Upsilon \left(P_{M},\;{\tilde{P}}_{\tilde{M}}\right)}{2}\right]{\left|S_{P_{M}}-\;{\tilde{S}}_{P_{M}}\right|}^{2\;}+\;\left[\frac{\Upsilon \left(P_{M},\;{\tilde{P}}_{\tilde{M}}\right)+N_{P_{M}}-\;{\tilde{N}}_{{\tilde{P}}_{M}}}{2}\right]{\left|S_{P_{M}}\right|}^{2\;}+\;\left[\frac{\Upsilon \left(P_{M},\;{\tilde{P}}_{\tilde{M}}\right)-N_{P_{M}}+\;{\tilde{N}}_{{\tilde{P}}_{M}}}{2}\right]{\left|S_{P_{M}}\right|}^{2\;}\\ \;\;\;if\;h_{l_{P}}\neq \;{\tilde{h}}_{\tilde{l_{P}}},\;h_{l_{S}}=\;{\tilde{h}}_{\tilde{l_{S}}}\;\\ \left[\frac{N_{P_{M}}+\;{\tilde{N}}_{{\tilde{P}}_{M}}-\;\Upsilon \left(P_{M},\;{\tilde{P}}_{\tilde{M}}\right)}{2}\right]{\left|S_{P_{M}}-\;{\tilde{S}}_{P_{M}}\right|}^{2\;}+\;\left[\frac{\Upsilon \left(P_{M},\;{\tilde{P}}_{\tilde{M}}\right)+N_{P_{M}}-\;{\tilde{N}}_{{\tilde{P}}_{M}}}{2}\right]{\left|S_{P_{M}}\right|}^{2\;}+\;\left[\frac{\Upsilon \left(P_{M},\;{\tilde{P}}_{\tilde{M}}\right)-N_{P_{M}}+\;{\tilde{N}}_{{\tilde{P}}_{M}}}{2}\right]{\left|\;{\tilde{S}}_{P_{M}}\right|}^{2\;}\\ \;\;\;\;\;+\left[\frac{N_{S_{\frac{M}{2}}}+\;{\tilde{N}}_{{\tilde{S}}_{\frac{M}{2}}\;}-\;\Upsilon \left(S_{\frac{M}{2}},\;{\tilde{S}}_{\frac{\tilde{M}}{2}}\;\right)\;}{2}\right]{\left|S_{S_{\frac{M}{2}}}-\;{\tilde{S}}_{S_{\frac{M}{2}}}\right|}^{2\;}+\left[\frac{\Upsilon \left(S_{\frac{M}{2}},\;{\tilde{S}}_{\frac{\tilde{M}}{2}}\;\right)+N_{S_{\frac{M}{2}}}-\;{\tilde{N}}_{{\tilde{S}}_{\frac{M}{2}}\;}\;}{2}\right]{\left|S_{S_{\frac{M}{2}}}\right|}^{2\;}+\left[\frac{\;\Upsilon \left(S_{\frac{M}{2}},\;{\tilde{S}}_{\frac{\tilde{M}}{2}}\;\right)-N_{S_{\frac{M}{2}}}+\;{\tilde{N}}_{{\tilde{S}}_{\frac{M}{2}}\;}\;}{2}\right]{\left|{\tilde{S}}_{S_{\frac{M}{2}}}\right|}^{2\;}\\ \;\;\;\;\;if\;h_{l_{P}}\neq \;{\tilde{h}}_{\tilde{l_{P}}},\;h_{l_{S}}\neq \;{\tilde{h}}_{\tilde{l_{S}}} \end{array}$$

### 4.2. Power Saving

One of the main underwater acoustic channel problems is its high transmission power requirement, which is up to tens of watts [5,6]. Unfortunately, such a problem reduces the lifetime of IoUT nodes, and is exacerbated by the limitations of their recharging capability. Such a problem can be solved by using an efficient modulation technique [25,26]. In this subsection, the saving in the power consumption of the proposed EFGSM scheme is considered in reference to the M-QAM modulation technique as in [15]. For more clarity, assume the needed data to be sent by IoUT node is at a rate of n bit/s/Hz. In the conventional case, this node will use the M-QAM modulation technique without the space modulation schemes (SMSs). However, by using the SMSs, the power will be saved by the reduced M dimension to the number of bits sent by SMSs. This saving ratio for SM, GSM, FGSM, and EFGSM schemes are given as follows, in reference to the M-QAM modulation:

(34) $$\eta_{SM}=1-\;\left(\frac{log_{2}\left(M\right)-\;log_{2}\left(N_{T}\right)}{log_{2}\left(M\right)}\right),$$

(35) $$\eta_{GSM}=1-\;\left(\frac{log_{2}\left(M\right)-\;log_{2}\left(N_{T}/N_{u}\right)}{log_{2}\left(M\right)}\right),$$

(36) $$\eta_{FGSM}=1-\;\left(\frac{log_{2}\left(M\right)-\;N_{T}+1}{log_{2}\left(M\right)}\right),$$

(37) $$\eta_{EFGSM}=1-\;\left(\frac{log_{2}\left(M\right)-\;log_{2}\left(M/2\right)\;-\;⎣log_{2}\sum_{k=2}^{N_{T}}\left(\begin{array}{c} N_{T}\\ k \end{array}\right)⎦}{log_{2}\left(M\right)}\right),$$

### 4.3. Computation Complexity Analysis

The receiver’s complexity of SMSs is addressed in this subsection based on the calculation of the TNRO for the ML decoders in each of the SMSs [16]. The receiver’s complexity is considered as a floating-point number (flops) which requires each decision of the ML decoder in terms of addition, subtraction, multiplication, division, and square root operations. The total number of flops is the TNCO. Therefore, in the conventional SM, the ML decoder considers the $\sum_{i=1}^{N_{r}}||y_{i}-\;h_{li}{S||}^{2}$ and needs a one combined multiplication (i.e., four actual multiplications) to be computed $h_{li}S$, and four real multiplications are required to calculate the square norm. In this way the conventional SM ML decoder uses this definition for calculating the TNCO, and its receiver’s complexity can be expressed as follows [16]:

(38) $$TNCO_{SM}=\;8N_{R}{\left(2\right)}^{R_{SM}}.$$

In the same way, the GSM receiver’s complexity is calculated. However, the way of calculating the $h_{li}$ in GSM is different than what is used in the conventional SM, where calculating $h_{li}$ of the GSM ML decoder requires $N_{T}$ − 1 complex summation with 2($N_{u}$ − 1) real summations [16,21]. Accordingly, the TNCO of the GSM ML decoder is expressed as follows [16]:

(39) $$TNCO_{GSM}=\;8N_{R}\left(2\left(N_{u}-1\right)\right){\left(2\right)}^{R_{GSM}}.$$

The maximum required complex summation in the ML decoder of the FGSM is $⎡\frac{N_{t}}{2}-1⎤$ for all $N_{t}\geq 3$. So, the TNCO of the ML decoder of the FGSM can be expressed as [16]:

(40) $$TNCO_{FGSM}=\;8N_{R}\left(2⎡\frac{N_{T}}{2}-1⎤\right){\left(2\right)}^{R_{FGSM}},$$

In the proposed EFGSM, to calculate the TNCO consider that there are two separate groups of antennas/combinations. First is the combination of the primary signal constellation and second is the combination of the secondary signal constellation. Both are similar to the FGSM, but each with different combinations. For these combinations, $y-\;h_{i}S_{j}$ are needed to be computed for the ML decoder of the primary and the secondary signal constellations, wherein the proposed EFGSM two symbols with two signal constellations are transmitted from two or more active transmitter antennas. Hence, the ML decoder computes those combinations based on (9). So, for all $N_{t}\geq 3,$ the signal transmitted from both the primary and secondary signal constellation needs at maximum $⎡\frac{N_{t}}{2}-1⎤$ complex summation. Therefore, The TNCO of the ML decoder of the proposed EFGSM can be given as:

(41) $$TNCO_{EFGSM}=\;8N_{r}\left[\left(\left(⎡2\frac{N_{T}}{2}-1⎤\right){\left(2\right)}^{R_{P}}\right)+\;\left(\left(2⎡\frac{N_{T}}{2}-1⎤\right){\left(2\right)}^{R_{S}}\right)\right],$$

where $R_{P}=log_{2}\left(P_{M}\right)$ and $R_{S}=log_{2}\left(S_{M/2}\right)$ represent the primary and secondary signal constellation rates respectively.

## 5. Simulation Results

In the following, computer simulation outcomes are presented for the proposed EFGSM over an uncorrelated underwater acoustic channel by using simulation outcomes achieved for $10^{6}$ symbols conveyed over a regular underwater acoustic channel as in [15,26]. In the receiver, the ML detection technique is used to estimate the transmitted symbols and indices and the average BER performances is obtained by the Monte Carlo simulation method using different transmission techniques. First, the proposed EFGSM achievable data rates are investigated at a different number of transmitted antennas, $N_{T}$, and compared with the conventional SM, GSM, and FGSM achievable data rates. Next, the ABER performance of the proposed scheme is evaluated by numerical and analytical simulations and compared with the current SMTs at various MIMO configurations. Then the power consumption efficiency and receiver computation complexity of the different SMTs are compared.

### 5.1. Achievable Data Rates

The highest achievable data rates in the case of infinite SNR conditions of the suggested EFGSM (6) are estimated following various number of $N_{T}$ and weighted upon the highest available data rates of SM, GSM, and FGSM given by (1), (2), and (4), respectively. Except when declared differently, all the supposed systems use 4-QAM modulation on their transmitter sides. Additionally, the actively transmitted antenna number $N_{T}$ in standard GSM is considered to be $N_{u}=2$. This value of active antenna, $N_{u}=2$, is maintained as increasing the $N_{u}$ degrades the current GSM BER performance, where increasing $N_{u}$ will increase the probability of having an equal antenna index in various antenna subsets [16].

**Figure 2:**
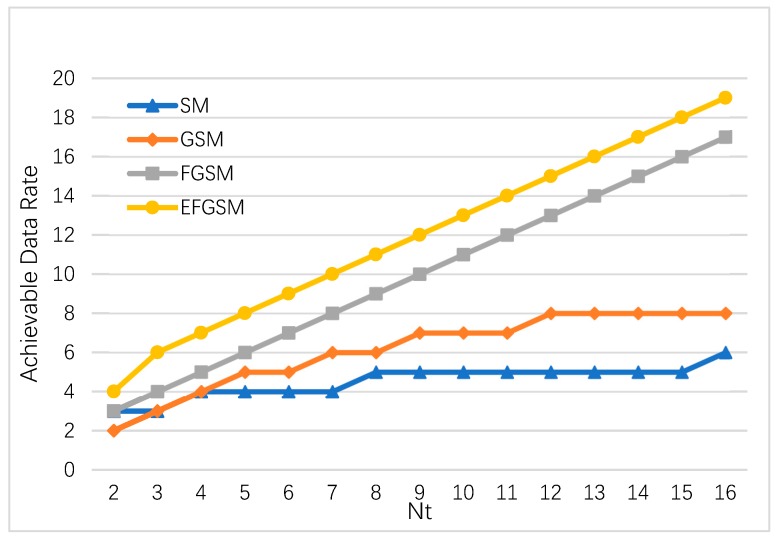
The maximum achievable data rates of the proposed EFGSM scheme compared to the maximum achievable data rates of the current SM, GSM, and FGSM at infinite SNR.

As shown in Figure 2, the proposed EFGSM outperforms the conventional SM, GSM, and FGSM in the available data rates where the available data rate in the suggested EFGSM increases linearly with *N_T_*. In SM, the available data rate increases logarithmically with $N_{T}$, whereas in GSM, it is increases logarithmically with the *N_T_* and *Nu*, combination. In the FGSM this increase is linear in regards to the *N_T_* − 1. For *N_T_* = 16, the proposed EFGSM achieves 19 (bpcu) compared to 6 (bpcu), 8 (bpcu) and 17 (bpcu) in case of SM, GSM and FGSM respectively. Consequently, the proposed schemes provide the highest SE, compared with low SE values given by the current SMTs.

### 5.2. The ABER Performance

**Figure 3:**
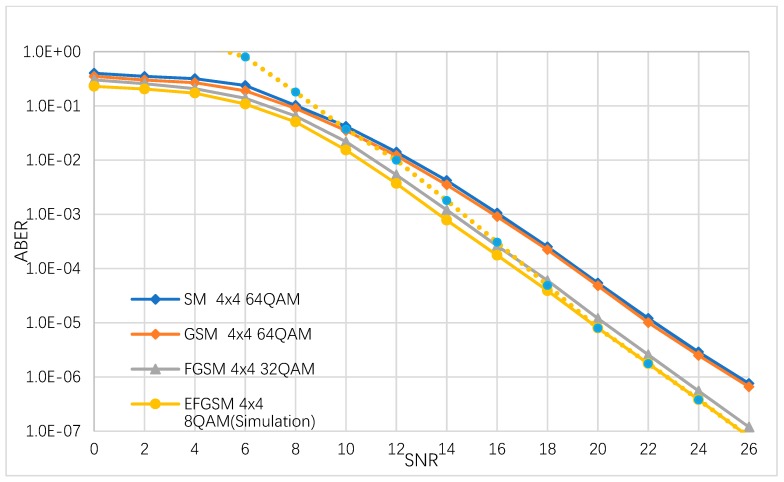
The ABER of the proposed EFGSM in comparison with the ABER of FGSM, GSM, and SM for 8 bpcu transmissions.

**Figure 4:**
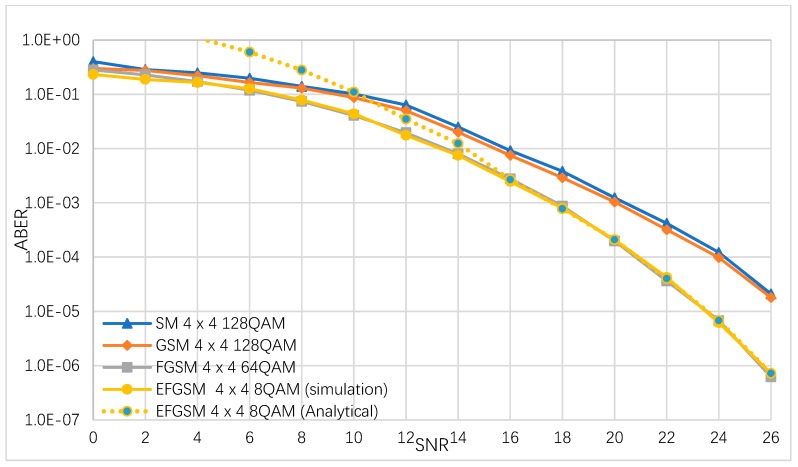
The ABER of the proposed EFGSM, FGSM, GSM and SM for 9 bpcu transmissions.

In this sub-section, the ABER performance of the suggested EFGSM is estimated by simulation numerically and analytically. We considered different modulation orders used to achieve 8 (bpcu) and 9 (bpcu) achievable rates. It is assumed that the same number of the transmitting and receiving (TX/RX) antennas are used in all schemes. The ABER of the proposed EFGSM compared to conventional SM, GSM and FGSM are depicted in Figure 3. The TX/RX antenna configuration assumed to be (4×4) in all schemes, and the MQAM modulation orders are 64/32/8 for (SM and GSM)/FGSM and the primary signal constellation of the proposed EFGSM, respectively. The BPSK is used as a secondary signal constellation of the proposed EFGSM. As shown in Figure 3, the proposed EFGSM outperforms the conventional SM, GSM, FGSM in terms of ABER. The ABER of achieving SE of 9 (bpcu) are depicted in Figure 4. Here as well, the TX/RX antenna configuration is assumed to be (4×4) in all schemes, and the M-QAM modulation orders are 128/64/8 for (SM and GSM)/FGSM and the primary signal constellation of the proposed EFGSM, respectively. In this case, the secondary signal constellation of the proposed EFGSM is quadrature phase shift keying (QPSK) modulation. Figure 4 shows that the EFGSM outperforms the conventional SM and GSM by 2.4 dB at nearly the same ABER of the conventional FGSM scheme. Based on that, the proposed EFGSM shows significantly better BER performance than current SM and GSM schemes and outperforms the performance of FGSM slightly. That allows the use of a lower modulation order than conventional SMSs. The FGSM has ABER performance nearly like that of the proposed EFGSM due to the varying of the antenna combinations (same data transmitted through more than one antenna providing high diversity with high power consumption).

### 5.3. Power Saving Evaluation

**Figure 5:**
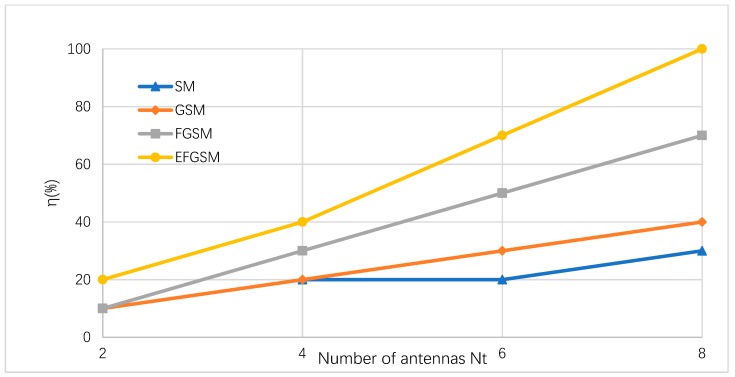
Power saving ratio of the proposed EFGSM at multiple Nt compared to conventional SM, GSM, and FGSM referenced to M (1024) QAM.

As shown in Section 4, to obtain the power efficiency of the proposed EFGSM, examine the claim that the IoUT sensor nodes are required to transfer data with a speed of 10 bit/s/Hz. In this case, without SMSs, suppose the IoUT node use M (1024) QAM. When using SMSs, there will be a decrease in M relationship to the number of bits which are sent by SMSs. Accordingly, power will be saved. The power saving ratios of SM, GSM, FGSM, and the proposed EFGSM are calculated in (34), (35), (36) and (37), respectively. The power saving ratio of different SMSs in respect to the number of transmitted antennas $N_{T}$ is shown in Figure 5, and for $N_{T}=4$, the proposed EFGSM saves up to 40% of the used power, while SM, GSM, and FGSM save up to 20%, 20%, and 30%, respectively.

### 5.4. Computation Complexity

**Figure 6:**
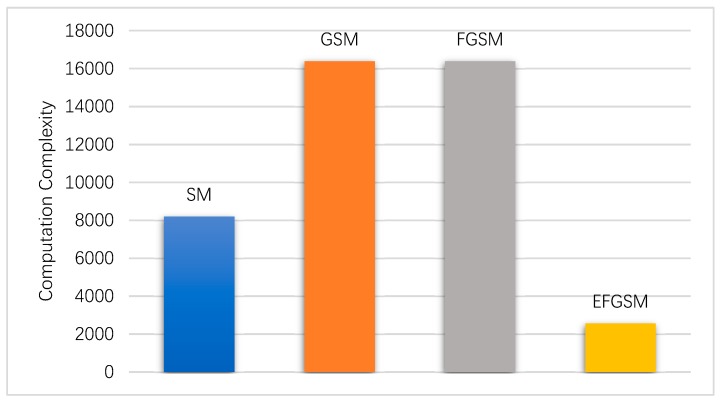
The computation complexity of the proposed EFGSM compared to the SM, GSM and FGSM computational complexity for 8 bpcu transmissions.

**Figure 7:**
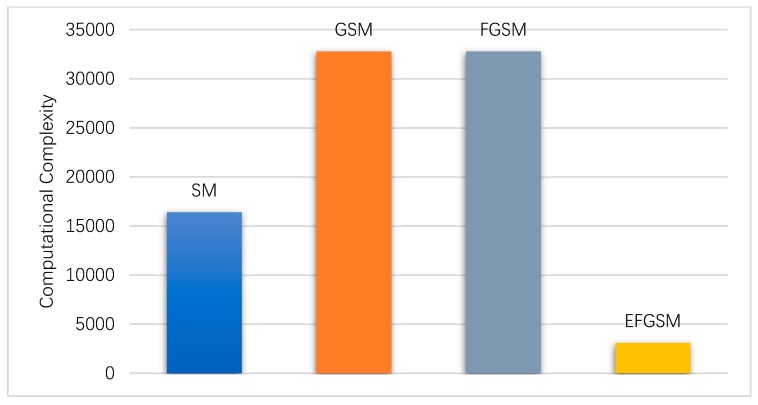
The computation complexity of the proposed EFGSM compared to the SM, GSM and FGSM computational complexity for 9 bpcu transmissions.

In this subsection, the computation complexity (i.e., TNRO) of the ML decoder used in the proposed EFGSM receiver side is calculated based on (38) and weighted toward the computation complexity of ML decoder used in the SM, GSM, FGSM receiver sides, respectively. As explained in Section 4, the proposed scheme receives two independent signals; the first one is related to the primary signal constellation, and the other is related to the second signal constellation. Each one is like the conventional FGSM in the TNRO and the complexity of the two signals (y_1_, y_2_) can be calculated as in (41). As in the ABER evaluation, in regards to achievable data rate, we calculate the system complexity based on, 8 and 9 bpcu. Assume the same TX/RX antennas are employed with different modulation orders in the different SMSs. Figure 6 and Figure 7 show the computational complexity reduction achieved by using the proposed scheme compared to the traditional SM, GSM, and FGSM. As shown, the computation complexity of the ML decoder in the receiver side is reduced significantly, as transmission through two different constellations provides an orthogonal signal which reduces the complexity of the ML decoder.

## 6. Conclusions

In this paper, a novel energy-efficient spatial modulation scheme called enhancement FGSM has been proposed for IoUT applications. The proposed scheme uses an innovative method to select the transmitter antennas to be activated for data transmission in two different data signal constellations in order to transmit additional data bits. More specifically, the suggested EFGSM is different from the current SMTs in terms of the process of choosing the transmitter antennas carrying information by primary signal constellation and secondary signal constellation. In the proposed scheme, for each case of primary signal constellation and secondary signal constellation, the number of active antennas may vary from the activation just a single transmitter antenna to the case of activating multiple transmitters. As a result, it achieves improvements in the ABER, spectral and power efficiency over the current SM, GSM, and FGSM spatial modulation schemes. The proposed scheme reduces the complex computation of the receiver by more than 90% over the conventional FGSM. The simulation and numerical results show the potential of EFGSM as an energy-efficient underwater communication system.

## Figures and Tables

**Table 1:** Example of EFGSM mapping table, in the case of 7 bpcu, 4-QAM modulation order and four antennas to transmit/receive.

Data Bits	Antennas Combination	Signal Constellation	Data Bits	Antennas Combination	Signal Constellation
0000	$T_{x1}T_{x2}$	$P_{M}:S_{M/2}$	1000	$T_{x1}T_{x2}$	$S_{M/2}:\;P_{M}$
0001	$T_{x1}T_{x3}$	$P_{M}:S_{M/2}$	1001	$T_{x1}T_{x3}$	$S_{M/2}:\;P_{M}$
0010	$T_{x1}T_{x4}$	$P_{M}:S_{M/2}$	1010	$T_{x1}T_{x4}$	$S_{M/2}:\;P_{M}$
0011	$T_{x2}T_{x3}$	$P_{M}:S_{M/2}$	1011	$T_{x2}T_{x3}$	$S_{M/2}:\;P_{M}$
0100	$T_{x2}T_{x4}$	$P_{M}:S_{M/2}$	1100	$T_{x2}T_{x4}$	$S_{M/2}:\;P_{M}$
0101	$T_{x3}T_{x4}$	$P_{M}:S_{M/2}$	1101	$T_{x3}T_{x4}$	$S_{M/2}:\;P_{M}$
0110	$T_{x1}T_{x2}\;\;T_{x3}$	$P_{M}:S_{M/2}:\;S_{M/2}$	1110	$T_{x1}T_{x2}\;\;T_{x3}$	$S_{M/2}:\;P_{M}:\;P_{M}$
0111	$T_{x1}T_{x2\;}T_{x4}$	$P_{M}:S_{M/2}:\;S_{M/2}$	1111	$T_{x1}T_{x2\;}T_{x4}$	$S_{M/2}:\;P_{M}:\;P_{M}$
